# Attitudes toward Medical Ethics among Obstetricians and Gynecologists in Saudi Arabia: An Exploratory Survey

**DOI:** 10.3390/healthcare11101394

**Published:** 2023-05-11

**Authors:** Ghiath Alahmad, Nuha Abed Althagafi

**Affiliations:** 1 King Abdullah International Medical Research Center, King Saud bin Abdulaziz University for Health Sciences, Riyadh 11481, Saudi Arabia; abedn@ngha.med.sa; 2 Department of Obstetrics and Gynecology, King Fahad National Guard Hospital, Riyadh 11426, Saudi Arabia

**Keywords:** obstetrics, gynecology, ethical principles, ethics training, ethical issues, Saudi Arabia

## Abstract

Ethics is an important aspect of medical care. The purpose of this study was to investigate the attitudes of obstetricians and gynecologists towards various ethical issues and ethical principles, and their satisfaction with their knowledge, understanding, and problem-solving skills regarding ethical issues. *Methods:* A cross-sectional survey was conducted among the working OB/GYNs in Saudi Arabia from various hospitals in Saudi Arabia between May 2020 and August 2020. A link to the three-point Likert scale questionnaire was mailed to 1000 OB/GYNs working in various hospitals. The data were analyzed using inferential statistics. The quantitative data were expressed as absolute numbers and percentages. *Results:* A total of 391 out of 1000 OB/GYNs responded. Most of the respondents were female OB/GYNs (65%), most of them were working in tertiary government hospitals (63%), and most were educated in bioethics (62%). About 80.3% of the respondents considered ethics important, and there was a low satisfaction rate with their knowledge (26%), understanding (38.6%), and problem-solving skills (35.8%) related to ethical issues. *Conclusions:* The obstetricians and gynecologists considered ethics an important aspect of daily practice but lacked the skills and knowledge to deal with ethical issues. The level of satisfaction with practice ethics was very low. Despite the fact that most of them had undergone bioethics education, most of them expressed the need for ethics training. Theoretical ethics education seemingly did not increase competence in resolving ethical issues, whereas experience did. The workplace had a strong correlation with the employee’s attitude toward ethical issues, principles, and satisfaction with their knowledge and skills in resolving ethical issues. The ethics curriculum needs to be structured in a more effective way to improve competence in dealing with ethical challenges in daily practice.

## 1. Introduction

Ethics is an important dimension of medical care and becomes even more distinct in obstetrics and gynecology, as the field not only deals with a single patient at a time but, in many cases, two—the mother and the fetus. Ethical competence is a key factor in decision-making. It helps obstetricians and gynecologists (OB/GYNs) find better solutions or alternative solutions whenever needed for the patients. Ethical competence improves the doctor–patient relationship [1]. In the early 1970s, the terms “fetal patient” and “fetus as a patient” entered the medical literature, signifying the importance of applying practice ethics to the fetus as well. The purpose of biomedical ethics is to study, build, and judge the criteria necessary to access complex situations and arrive at a solution that is both medically and ethically correct [2]. Autonomy, beneficence, non-maleficence, and justice are the primary ethical principles that were proposed by Beauchamp and Childress in 1979 and which have been implemented over the past 50 years [3].

Ethical dilemmas related to obstetrics and gynecology may also be due to age-old practices such as female genital mutilation, consanguineous marriages, adolescent marriage and pregnancy, and the termination of pregnancy for medical and non-medical reasons, or due to modern technologies such as sex selection, choice of delivery methods, conception methods, surrogacy, egg donations [4], prenatal diagnosis [5], selective abortions [6], and the use of genetic information [7]. In these situations, medical knowledge alone will potentially not enhance the decision-making skills. A pluralistic society has many sources of morality, and medical ethics should seek to bridge these differences. An ethically aware physician should identify and understand his or her obligations towards their patients, irrespective of ethnic and cultural variations [2].

Other principles found in modern ethical theories include virtues (virtues of integrity, humility, compassion, self-efficacy, and self-sacrifice), ethics of care, feminist ethics, and communitarian ethics [8,9]. Autonomy, beneficence, non-maleficence, and justice are also the principles of Islamic bioethics. Islamic bioethics is a system of moral assessment primarily based on the principles of the Qur’an and Sunnah that focuses on analyzing and solving the ethical issues that arise in medical practice and research [10,11,12,13,14,15].

The ethics of OB/GYNs are not only involved in terms of patients, treatment, and care, but also in the responsibility of the physicians to care about future generations and the environment. The WHO claims that 10% of medical waste is infectious or highly infectious, 5% consists of toxic chemicals or is radioactive or carcinogenic, and 15% is hazardous [16]. There are various hazardous wastes generated within obstetrics and gynecology hospitals that pose a serious risk to the environment. In Saudi Arabia, the estimated average of all healthcare-related waste generated per year is 25,207 tons [17]. According to the report [18], the amount of hazardous waste generated by a Women’s Wellness and Research Center in Qatar between 2017 and 2019 was 344,755 kg. This included infectious waste, sharp waste, pathological waste, and cytotoxic waste. Medical waste management has become a serious issue as the waste poses dangerous threats to the environment. Most biomedical waste is non-biodegradable and can cause a long-lasting impact on the environment.

This study aimed to explore the attitudes of OB/GYNs toward their practice ethics and various primary and secondary ethical principles. The survey was conducted among OB/GYNs belonging to different hospitals. Our analysis addressed the fact that although the OB/GYNs emphasized the importance of ethics, their practicability and satisfaction were quite questionable.

## 2. Materials and Methods

### 2.1. Sample Size

The study sample included obstetricians and gynecologists currently working in various hospitals throughout the Kingdom of Saudi Arabia. The participants, who belonged to different age groups and nationalities and had different years of experience, were anonymously and randomly selected after their coded contact details were received from the Saudi Society of OB/GYN. Monkey surveys (e-mails, social media, WhatsApp, Facebook) were used as the questionnaire delivery method, and reminder emails were sent three times a week. The link to the questionnaire was randomly sent to 1000 OB/GYNs, out of whom 391 responded. 

### 2.2. Study Design and Setting

The survey was conducted between May 2020 and August 2020. The email addresses of the OB/GYNs working in various hospitals were collected. An introductory email explaining the nature of the survey, along with the link to the questionnaire, was mailed to the selected participants. The questionnaire contained three segments, each with a set of 24 multiple-choice questions representing a different topic. The first part was designed to acquire the demographic data of the participants, and the second part was for acquiring information about the participants’ attitudes towards ethics in daily practice. The next segment of the questionnaire was a self-contemplation about skill, knowledge, and understanding of ethics in daily practice, and the last segment had questions related to the primary and secondary bioethical principles. The validity of the questionnaires used in the study was ensured by a team of experts. A pilot study of five subjects was conducted in order to ensure test–retest reliability before formal data collection commenced. Cronbach’s alpha was used to measure the consistency coefficient of the questionnaire, which achieved a high score of 0.87. No incentive was given for participating in this research.

The responses were limited to a 3-point Likert scale, where the options were yes, do not know, and no. The participants had to choose any one of the three given options.

### 2.3. Statistical Methods

The data were de-identified after the responses were obtained and analyzed using inferential statistics. Subsequently, the respondents were grouped according to their demographic characteristics and their responses to various questions. The quantitative data were expressed as absolute numbers and percentages. To examine the relationship between OB/GYN and predictor variables, *t*-tests and ANOVA were used. The relationship between the dependent and independent variables was analyzed. For all associations, *p* < 0.05 was considered significant.

### 2.4. Ethical Considerations

An official approval for conducting this study was obtained from the IRB at King Abdulaziz Medical City. Following this, participants were contacted and asked about their willingness to participate in our study. They received a brief explanation, and their written consent was sought. Confidentiality and privacy were respected, and identical information was collected. Participants were advised to contact the primary investigator by email or phone for any further clarifications or inquiries.

## 3. Results

### 3.1. Demographic Characteristics of Respondents

**Figure 1:**
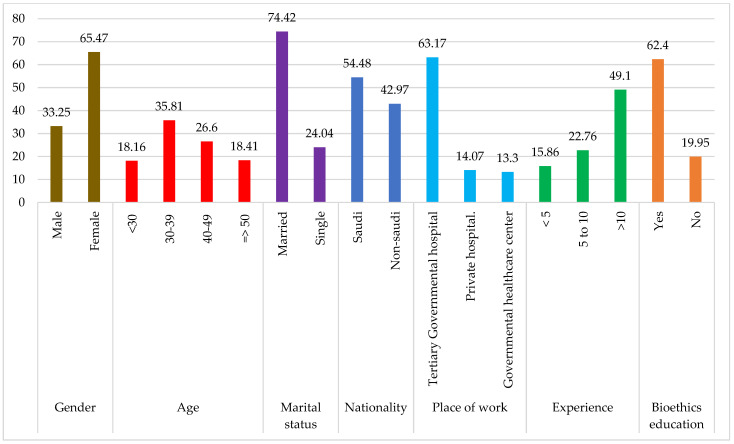
Demographics Chart of Respondents.

A total of 391 OB/GYNs responded, out of which 130 (33.25%) male OB/GYNs and 256 (65.47%) female OB/GYNs (a total of 98.72%) completed the full set of questionnaires. The respondents belonged to different age groups, from less than 30 (18.16%) to equal to or more than 50 years (18.41%) of age. The highest number of respondents were in the age group 30–49 (62.4%). Most of the respondents were married (74.42%). The survey included both Saudi citizens (54.48%) and non-citizens (42.97%). The respondents worked in either of three types of hospitals, tertiary government hospitals (63.17%), private hospitals (14.07%), or government healthcare centers (13.30%), with a wide range of experience. Most of the respondents had more than 10 years of experience (49.10%). A total of 15.86% and 22.76% of respondents had under 5 years and 5–10 years of experience, respectively. Most of the respondents were educated in bioethics (62.40%), and about 19.95% of the participants did not have bioethics education. The response rate varied for each question and each demographic characteristic. The demographic data of the respondents and their response rates are given in Table 1 and represented in Figure 1.

### 3.2. Attitude towards Various Practice Ethics

**Figure 2:**
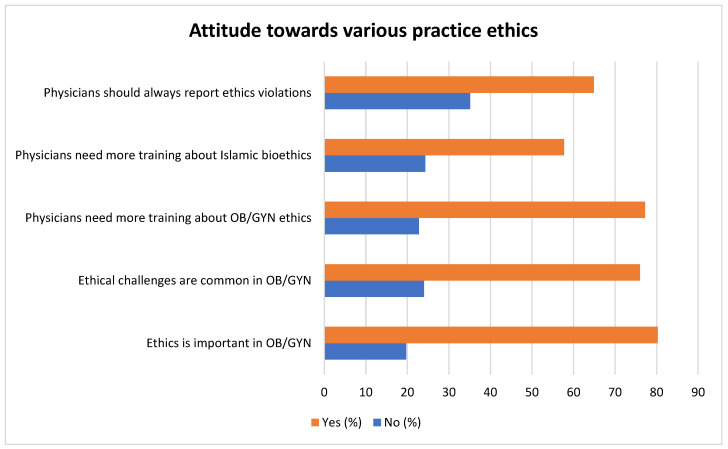
Attitude of OB/GYNs towards various practice ethics.

The attitudes of OB/GYNs towards practice ethics are represented in Figure 2. About 80.3% of the respondents considered ethics important, and 76% considered ethical challenges common in the field of obstetrics and gynecology. A total of 77.2% of the respondents accepted that they needed more training in ethics, and 64.9% considered that ethical violations should be reported.

The associations between attitudes toward various ethical statements and the sociodemographic variables are depicted in Table 2. 

In general, the female OB/GYNs had a more positive attitude towards the practice ethics. The respondents aged below 30 had the lowest rate of positive responses, while those aged 30 to 49 had the highest rate of agreement. This rate declined in the age group above 49 years. To our surprise, this trend was similar across all the ethical statements. Most of the time, marital status did not have any significance on the attitude towards the ethical statements. When compared, the citizens of Saudi Arabia had the highest percentage of positive responses compared to the non-citizens for all the statements. The association between nationality and the statement “Physicians should always report ethics violations” was statistically significant (*p* = 0.010). The positive response rate for all the ethical statements was low in the respondents from healthcare centers and was mostly high in private hospitals. The relationship between the workplace and the statements that ethics is important in OB/GYN (*p* = 0.003), ethical challenges are common in OB/GYN (*p* = 0.000), and physicians should always report ethics violations (*p* = 0.003) approached significance. The associations between bioethics education and the ethical statements that physicians need more training about OB/GYN ethics (*p* = 0.043) and physicians need more training about Islamic bioethics (*p* = 0.024) were statistically significant. The trend seen in years of experience was somewhat similar to the age group. Respondents with less than 5 years of experience had a lower rate of positive response, while respondents with 5–10 years of experience had the highest rate of positive response for the majority of the statements. 

### 3.3. Self-Contemplation about Practice Ethics and Ethical Challenges

**Figure 3:**
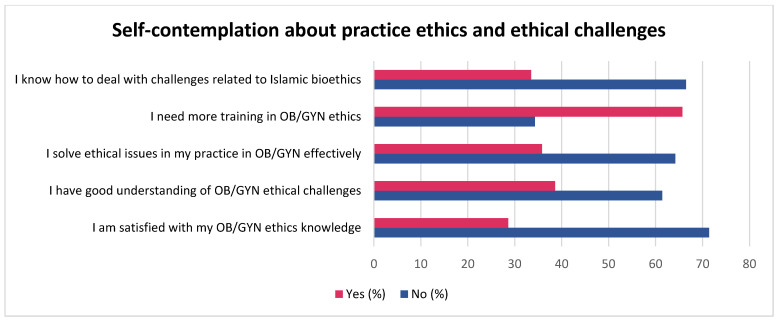
Self-contemplation of OB/GYNs about practice ethics and ethical challenges.

The responses to various self-contemplation statements on practice ethics are given in Figure 3. Though most of the OB/GYNs considered ethics important, they had lower satisfaction with their knowledge (28.6%), skills (35.8%), and understanding (38.6%). About 65.7% of the OB/GYNs expressed the need for more ethics training.

The association between self-contemplation and the sociodemographic variables is depicted in Table 3. 

Despite the high number of female respondents, only 24% of the female OB/GYNs were satisfied with their ethical knowledge. About 69.60% of female and 58.50% of male respondents expressed that they needed more training in OB/GYN ethics. The associations between age and satisfaction (*p* = 0.000), understanding (*p* = 0.000), issue-solving skill (*p* = 0.001), the need for training in ethics (*p* = 0.028), and knowledge about Islamic bioethics (*p* = 0.010) were statistically significant. About 64% of respondents below 30 years of age had a positive response about their problem-solving skills. About 70% of the respondents who belonged to the age group of 30–49 years expressed that they need more training in OB/GYN ethics. Unlike the attitude towards practice ethics, to our surprise marital status had a strong significance for all the statements (*p* = 0.000; 0.000; 0.000; 0.000; and 0.013). Non-citizens had a high positive response for satisfaction, understanding, problem-solving skills, and knowledge about Islamic bioethical principles, while most of the citizens expressed the need for training in OB/GYN ethics. The relationship between nationality and satisfaction with knowledge (*p* = 0.053), understanding (*p* = 0.013), problem-solving skills (*p* = 0.049), and skills regarding Islamic bioethics (*p* = 0.015) approached significance. Statistical significance was observed in the relationship between workplace and satisfaction (*p* = 0.000), understanding (*p* = 0.001), and problem-solving skills (*p* = 0.001). To our surprise, bioethics education did not seem to have a positive impact on the attitude of the OB/GYNs. About 80% of the respondents who had prior bioethics education felt that they needed more training on OB/GYN ethics. The years of experience were statistically significant across all the statements (*p* = 0.000; 0.000; 0.001; 0.039; and 0.002).

### 3.4. Attitude towards Primary and Secondary Ethical Principles

**Figure 4:**
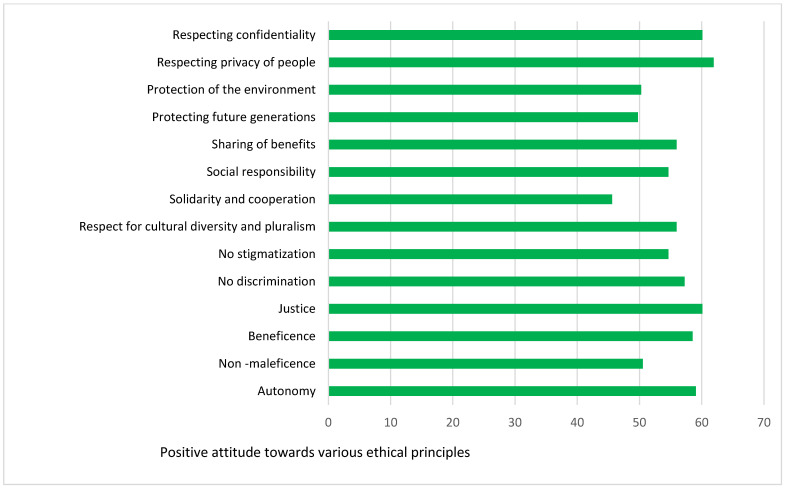
Attitude towards ethical principles.

The attitude towards primary and secondary ethical principles is represented in Figure 4. The respondents had a higher positive attitude for almost all the primary and secondary ethical principles, except for protecting the environment, protecting future generations, and solidity and cooperation.

The association between the primary and secondary ethical principles and sociodemographic variables is depicted in Table 4, Table 5 and Table 6. 

The rates of positive attitude towards almost all the ethical principles were the same in both male and female OB/GYNs, except for justice (M = 61% and F = 71%), protecting future generations (M = 55% and F = 46%), and protecting the environment (M = 54% and F = 48%). When compared between age groups, the positive attitude was higher in those under 30 years of age for the principles of beneficence, solidarity and cooperation, social responsibility, sharing of benefits, protection of the environment, and respecting confidentiality. The 30–39 age group had a positive attitude towards autonomy, non-maleficence, justice, no discrimination, protecting future generations, and respecting people’s privacy. However, the above-mentioned 49-year-old age group had the least positive attitude across all the ethical principles. We observed that as the age increased, the positive attitude towards ethical principles decreased. The relationship between nationality and sharing of benefits (*p* = 0.041) and respecting the privacy of people (*p* = 0.02) was statistically significant. The workplace was statistically significant across all the ethical principles except for protecting future generations (*p* = 0.027; *p* = 0.013; *p* = 0.008; *p* = 0.013; *p* = 0.014; *p* = 0.007; *p* = 0.006; *p* = 0.032; *p* = 0.024; *p* = 0.045; *p* = 0.012; *p* = 0.007; *p* = 0.048). Respondents without prior bioethics education had a highly positive attitude towards most of the ethical principles, whereas respondents who had received bioethics education had a positive attitude only towards non-maleficence.

## 4. Discussion

In this study, we investigated the OB/GYNs’ attitudes towards various practice ethics and ethical principles. In most of the cases, the female respondents had a high degree of positive attitude towards medical practice ethics, conveying the importance of ethics. Despite this, only a very few female respondents were satisfied with their knowledge, understanding, and problem-solving skills related to practice ethics and Islamic bioethics, and ultimately expressed the need for more training in ethics. There was a correlation between age group and years of experience in attitudes towards the practice of ethics. The respondents who belonged to the age group below 30 and had experience below 5 years had the lowest rate of positive attitude, while the respondents in the mid-age group (30–49) and those with 5–10 years of experience had a high rate of positive attitude for almost all the statements related to ethics. There was a steady decline in this attitude among the respondents below 49 years. The correlation between age group and years of experience also existed regarding satisfaction, understanding, problem-solving skills, and knowledge. 

Though there was not much influence of marital status on attitudes toward practice ethics, it had a high impact on satisfaction with practice ethics. Most of the married respondents had a high degree of satisfaction with various practice ethics and little need for OB/GYN ethics training. It can be hypothesized that this may be an outcome of dual-physician marriages. It has been reported that there is an increased satisfaction related to the job in medical marriages. In medical marriages, the physicians have the scope of discussing various issues with the spouse and finding solutions to problems [19]. To our surprise, the non-citizens of Saudi Arabia had a more positive response to Islamic bioethics than the native OB/GYNs. Reference [20] states that patients’ rights, equity of resources, the confidentiality and safety of patients, conflicts of interest, healthcare team ethics, Islamic medicine, and the ethics of Muslim doctors are the major medical ethical challenges in Saudi Arabian healthcare. It has been observed that there is no difference between the Western teaching of medicine, which most of the doctors have undergone, and the Islamic practice of medicine.

Respondents from private hospitals expressed the need for ethics training, but also expressed satisfaction with their knowledge, understanding, and problem-solving skills. Though the respondents with bioethics education had a comparatively higher positive attitude towards practice ethics, a significant number of respondents without bioethics education also had a positive attitude towards ethics in daily practice. However, neither of these groups of respondents were satisfied with their knowledge, understanding, and problem-solving skills, and the need for more training in OB/GYN ethics was higher in respondents with prior bioethics education. Though there is an increasing need for and importance of bioethics education in Saudi society, the guidance on teaching and learning bioethics is limited. Bioethics theoretical education lacks a structured curriculum and does not provide proper guidance to address the ethical dilemmas faced in daily practice [12,20]. Survey [21] reports that out of 191 OB/GYNs, 42% of them felt unprepared to deal with ethical challenges in daily practice. This is in line with our survey, as respondents with prior bioethics education expressed the need for more training in bioethics. Ethics education can be improved by including hands-on training, case-based studies in the curriculum, and informal discussion with the faculties. Survey [22], among family medicine trainees in Saudi, states that about 93.8% of the trainees were unaware of the Helsinki declaration of medical ethics, and the trainees’ understanding, attitudes, and practices regarding medical ethics were deficient.

Every branch of medicine has an ethical dilemma of its own, and the field of OB/GYNs is distinct as it deals with the mother and unborn child. The ethical dilemma arises in situations where the OB/GYNs face the decision to prioritize either the health of the mother or the fetus. Professional ethical concern is inversely proportional to job satisfaction [23]. Survey [24] reports that 32% (N = 220) of healthcare professionals were not completely aware of the laws pertaining to the rights of the fetus. Abortion is one of the common forms of ethical dilemmas faced by obstetricians. According to Islamic bioethics, abortion is forbidden and can be carried out only if the mother’s life is at risk or if the fetus has anomalies that are incompatible with life. Even in such cases, abortion is permissible only before the 120th day of gestation [25]. Saudi law is consistent with this legal fatwa, by prohibiting and criminalizing abortion [14]. Hence, doctors in Saudi who oppose performing abortions do not suffer from a conscientious moral dilemma because there is no contradiction between the text of the law and the rule of Sharia on the one hand, and their moral conscience and their professional duty on the other hand. This is unlike what happens in many countries when doctors who oppose abortion for ethical considerations find themselves opposing the law that permits abortion. This ethical dilemma has raised concern in many countries. Recently, a law was passed in Italy in favor of conscientious objection to abortion. The law states that the physician can refuse to perform an abortion on conscience grounds but is still obligated to serve the patient before and after the procedure [26]. This is despite the existence of a law that gives women the right to abortion [27]. In a survey in Poland, about 6% (N = 71) of gynecologists stated that they would never perform abortions because it was against their consciences [28].

Ethical unawareness and a lack of competence could lead to negligence and increased litigation rates. In a survey among 826 OB/GYNs in Australia, only 44% intended to continue obstetrics after 5 years. Fear of litigation was one of the main reasons stated to discontinue obstetrics practice [29]. In a survey study [30] during the period 2008–2016, it was observed that malpractice claims for obstetrics and gynecology accounted for about 24% of the overall malpractice claims in Saudi Arabia. According to the American Medical Association, a total of 34% of the obstetricians had faced litigation.

Both the male and female participants had positive attitudes towards both primary and secondary ethical principles. However, ethical principles such as autonomy can be seen with different perceptions in Saudi society compared to the Western world. This is because the freedom to make decisions is restricted not only by law, but also by Sharia, without causing a contradiction because the legal system in Saudi Arabia is based on Islamic law, also known as Sharia. This means that all laws must be in accordance with the teachings of the Qur’an and Sunnah [31]. Women’s freedom of choice may be constrained by traditional values and customs, which applies to health care, especially with regard to giving informed consent. However, the rate of positive response was low in the age group above 49 for all the ethical principles. Marital status and years of experience did not have much influence on the attitude, whereas among the nationality group the citizens had a stronger positive attitude towards all the ethical principles than the non-citizens. When compared among workplaces, the governmental health care centers had a significantly lower positive attitude towards all the ethical principles. Bioethics education did not have much influence on attitude. In fact, in certain cases (social responsibility and sharing of benefits), the positive attitude was higher in respondents without bioethics education.

When comparing the demographic data, it can be observed that respondents from every demographic group considered ethics important and had a lower positive attitude towards the statement “Physicians Should Always Report Ethics Violations”. A very low percentage of respondents were satisfied with their ethics knowledge, and the need for ethics training was significantly high across all demographic groups. When compared among ethical principles, the positive attitude was higher towards respecting privacy, respecting confidentiality, justice, beneficence, and autonomy, and lower towards solidarity and cooperation, non-maleficence, protecting the environment, and protecting future generations. It is important to increase awareness among OB/GYNs about their attitudes toward the environment. It has become imperative for OB/GYNs to optimize the health benefits of implementing environmentally sustainable practices. A “greener” approach to obstetrics care contributes to the sustainable future of women and children. OB/GYNs should try to implement environmentally safe practices as much as possible. The use of digital medical records is one way of implementing “greener” health care [32]. The mean values across the demographic data are given in Table 7.

### Study Limitations

A relatively small sample size can be considered one of the limitations of this study. There is a possibility of selection bias as the response rate was only 39%. Approximately 60% of the selected participants did not respond, which may have a strong influence on the survey results. The non-respondents may have had a different opinion about the various ethical statements. Some ethical principles may be understood differently by participants compared to the Western world, such as autonomy, which can have an impact on the generalization of the study’s findings. More in-depth research into the discussed area is essential.

## 5. Conclusions

It can be concluded that the workplace has a strong correlation with attitudes and satisfaction. Both bioethics education and experience were statistically significant in influencing attitudes towards ethical issues, but only experience had a correlation with satisfaction. This can be reasoned out as ethics education does not seemingly increase ethical competence, and only experience does. Gender, age, marital status, workplace, nationality and experience were all statistically significant to satisfaction. In the modern era, ethics cannot be limited only to autonomy, beneficence, non-maleficence, and justice, but must also recognize the importance of equity, patients’ privacy, pluralism, and having responsible behavior towards sustaining a greener environment and thus protecting future generations. The ethics curriculum needs to be structured in a more effective way to improve competence to deal with ethical challenges in daily practice. The awareness of various practical ethics and ethical principles is very important as this enhances the skills to face and address an ethical issue. However, knowledge and understanding of the ethical theories varies greatly among OB/GYNs. OB/GYNs who are familiar with ethics are able to handle complex ethical situations in a better way.

## Figures and Tables

**Table 1:** Demographic Table (N = 391).

Demographic Characteristics		Total No. of Respondents Who Completed the Questionnaire		Total Percentage of Respondents Who Completed the Questionnaire across Various Factors
		N	%	%
Gender	Male	130	33.25	98.72
	Female	256	65.47	
Age	<30	71	18.16	98.98
	30–39	140	35.81	
	40–49	104	26.60	
	≥50	72	18.41	
Marital status	Married	291	74.42	98.47
	Single	94	24.04	
Nationality	Saudi	213	54.48	97.44
	Non-Saudi	168	42.97	
Place of work	Tertiary governmental hospital	247	63.17	90.54
	Private hospital	55	14.07	
	Governmental healthcare center	52	13.30	
Experience	<5	62	15.86	87.72
	5–10	89	22.76	
	>10	192	49.10	
Bioethics education	Yes	244	62.40	82.35
	No	78	19.95	

**Table 2:** Attitudes (those who opted yes) towards various statements about OB/GYN practice ethics across demographic data.

Demographic Characteristics		Ethics is Important in OB/GYN		Ethical Challenges Are Common in OB/GYN		Physicians Need More Training about OB/GYN Ethics		Physicians Need More Training about Islamic Bioethics		Physicians Should Always Report Ethics Violations	
		N	%	N	%	N	%	N	%	N	%
Total		314	80.3	297	76.0	302	77.2	296	57.7	254	64.9
Sex	M	98	75.40	93	71.50	92	70.80	91	70.00	84	64.60
	F	214	83.30	202	78.60	208	80.90	203	79.00	165	64.20
		*p* = 0.064		*p* = 0.123		*p* = 0.024		*p* = 0.051		*p* = 0.936	
Age (years)	<30	51	71.80	51	71.80	50	70.40	48	67.60	42	59.20
	30–39	119	85.00	111	79.30	115	82.10	111	79.30	89	63.60
	39–49	86	82.70	82	78.80	82	78.80	81	77.90	71	68.30
	>49	57	79.20	52	72.20	54	75.00	55	76.40	49	68.10
		*p* = 0.130		*p* = 0.472		*p* = 0.245		*p* = 0.285		*p* = 0.579	
Married	Yes	234	80.40	220	75.60	224	77.00	220	75.60	182	62.50
	No	76	80.10	73	77.70	75	79.80	73	77.40	65	69.10
		*p* = 0.163		*p* = 0.392		*p* = 0.116		*p* = 0.063		*p* = 0.408	
Nationality	Saudi	174	81.70	170	79.80	170	79.80	164	77.00	150	70.40
	Others	135	80.40	123	73.20	127	75.60	127	75.60	97	57.70
		*p* = 0.741		*p* = 0.129		*p* = 0.324		*p* = 0.749		*p* = 0.010 *	
Workplace	Tertiary govern. hospital	224	90.70	216	87.40	213	86.20	208	84.20	187	75.70
	Private hospital	51	92.70	48	87.30	50	90.90	49	89.10	37	67.30
	Govern. healthcare center	39	75.00	33	63.50	39	75.00	39	75.00	27	51.90
		*p* = 0.003 *		*p* = 0.000 *		*p* = 0.074		*p* = 0.165		*p* = 0.003 *	
Bioethics education	Yes	237	97.18	223	91.17	228	93.62	225	92.45	188	77.78
	No	73	93.60	70	89.7	70	89.7	68	87.2	59	75.6
		*p* = 0.066		*p* = 0.093		*p* = 0.043 *		*p* = 0.024 *		*p* = 0.057	
Years of experience	<5	52	83.90	49	79.00	50	80.60	48	77.40	37	59.70
	5–10	81	91.00	77	86.50	79	88.80	76	85.40	61	68.50
	>10	175	91.10	165	85.90	166	86.50	165	85.90	146	76.00
		*p* = 0.234		*p* = 0.368		*p* = 0.354		*p* = 0.262		*p* = 0.039 *	

* Independent samples *t*-test/ANOVA is significant at α = 0.05.

**Table 3:** Self-contemplation about practice ethics and ethical challenges across demographic data.

Demographic Characteristics		I Am Satisfied with My OB/GYN Ethics Knowledge		I Have Good Understanding of OB/GYN Ethical Challenges		I Solve Ethical Issues in My Practice in OB/GYN Effectively		I Need More Training in OB/GYN Ethics		I Know How to Deal with Challenges Related to Islamic Bioethics	
		N	%	N	%	N	%	N	%	N	%
**Total**		112	28.6	151	38.6	140	35.8	257	65.7	131	33.5
Sex	M	48	36.90	66	50.80	61	46.90	76	58.50	55	42.30
	F	64	24.90	83	32.30	77	30.00	179	69.60	75	29.20
		*p* = 0.000 *		*p* = 0.000 *		*p* = 0.001 *		*p* = 0.028 *		*p* = 0.010 *	
Age (years)	<30	8	11.30	10	14.10	46	64.80	10	14.10	13	18.30
	30–39	36	25.70	50	35.70	45	32.10	106	75.70	43	30.70
	39–49	37	35.60	51	49.00	49	47.10	67	64.40	40	38.50
	>49	31	43.10	39	54.20	36	50.00	37	51.40	34	47.20
		*p* = 0.000 *		*p* = 0.000 *		*p* = 0.000 *		*p* = 0.005 *		*p* = 0.002 *	
Married	Yes	99	34.00	133	45.70	124	42.60	183	62.90	110	37.80
	No	10	10.60	15	16.00	14	14.90	72	76.60	20	21.30
		*p* = 0.000 *		*p* = 0.000 *		*p* = 0.000 *		*p* = 0.000 *		*p* = 0.013 *	
Nationality	Saudi	53	24.90	71	33.30	68	31.90	142	66.70	61	28.60
	Non-Saudi	57	33.90	77	45.80	70	41.70	111	66.10	68	40.50
		*p* = 0.05 *		*p* = 0.013 *		*p* = 0.049 *		*p* = 0.903		*p* = 0.015 *	
Workplace	Tertiary govern. hospital	72	29.10	102	41.30	91	36.80	182	73.70	84	34.00
	Private hospital	29	52.70	32	58.20	30	54.50	39	70.90	29	52.70
	Govern. healthcare center	11	21.20	17	32.70	19	36.50	36	69.20	18	34.60
		*p* = 0.000 *		*p* = 0.001 *		*p* = 0.001 *		*p* = 0.755		*p* = 0.069	
Bioethics education	Yes	89	37.17	118	48.32	102	41.97	195	80.04	102	42.83
	No	21	26.9	30	38.5	35	44.9	59	75.6	28	35.90
		*p* = 0.171		*p* = 0.506		*p* = 0.808		*p* = 0.549		*p* = 0.167	
Years of experience	<5	12	19.40	14	22.60	17	27.40	46	74.20	12	19.40
	5–10	20	22.50	31	34.80	26	29.20	74	83.10	28	31.50
	>10	80	41.70	103	53.60	93	48.40	132	68.80	87	45.30
		*p* = 0.000 *		*p* = 0.000 *		*p* = 0.001 *		*p* = 0.039 *		*p* = 0.001 *	

* Independent samples *t*-test/ANOVA is significant at α = 0.05.

**Table 4:** Attitude towards primary ethical principles across demographic data.

Characteristics		Autonomy		Non-Maleficence		Beneficence		Justice	
		N	%	N	%	N	%	N	%
Sex	M	76	58.46	66	50.77	73	56.15	80	61.54
	F	152	59.38	129	50.39	153	59.77	182	71.09
	*p* value	0.897		0.915		0.524		0.65	
Age (years)	<30	42	59.15	37	52.11	47	66.20	44	61.97
	30–39	86	61.43	77	55.00	85	60.71	88	62.86
	39–49	62	59.62	52	50.00	61	58.65	64	61.54
	>49	39	54.17	30	41.67	35	48.61	37	51.39
	*p* value	0.717		0.279		0.144		0.357	
Married	Yes	170	58.42	144	49.48	165	56.70	176	60.48
	No	55	58.51	47	50.00	58	61.70	54	57.45
	*p* value	0.938		0.525		0.561		0.784	
Nationality	Saudi	134	62.91	115	53.99	133	62.44	132	61.97
	Others	93	55.36	80	47.62	94	55.95	99	58.93
	*p* value	0.136		0.217		0.2		0.546	
Workplace	Tertiary govern. hospital	152	61.54	128	51.82	151	61.13	151	61.13
	Private hospital	32	58.18	29	52.73	33	60.00	34	61.82
	Govern. healthcare center	24	46.15	20	38.46	22	42.31	24	46.15
	*p* value	0.027 *		0.013 *		0.008 *		0.013 *	
Bioethics education	Yes	142	58.20	127	52.05	142	58.20	145	59.43
	No	50	64.10	37	47.44	48	61.54	47	60.26
	*p* value	0.628		0.594		0.878		0.714	
Years of experience	<5	36	58.06	33	53.23	38	61.29	41	66.13
	5–10	56	62.92	50	56.18	57	64.04	53	59.55
	>10	110	57.29	90	46.88	105	54.69	109	56.77
	*p* value	0.655		0.278		0.26		0.375	

* Independent samples *t*-test/ANOVA is significant at α = 0.05.

**Table 5:** Attitude towards secondary ethical principles (Part A) across demographic data.

Characteristics		No Discrimination		No Stigmatization		Respect for Cultural Diversity and Pluralism		Solidarity and Cooperation		Social Responsibility	
		N	%	N	%	N	%	N	%	N	%
Sex	M	72	55.4	71	54.6	74	56.9	60	46.2	73	56.2
	F	149	58.2	140	54.7	142	55.5	116	45.3	138	53.9
	*p* value	0.627		0.979		0.755		0.849		0.647	
Age (years)	<30	41	57.8	38	53.5	40	56.3	35	49.3	42	59.2
	30–39	87	62.1	81	57.9	79	56.4	65	46.4	77	55
	39–49	60	57.7	61	58.7	63	60.6	51	49	57	54.8
	>49	34	47.2	32	44.4	35	48.6	26	36.1	36	50
	*p* value	0.207		0.26		0.503		0.29		0.641	
Married	Yes	165	56.7	158	54.3	162	55.7	127	43.6	154	52.9
	No	53	56.4	51	54.3	53	56.4	47	50	56	59.6
	*p* value	0.786		0.832		0.749		0.349		0.438	
Nationality	Saudi	124	58.2	120	56.3	124	58.2	104	48.8	125	58.7
	Others	95	56.6	91	54.2	92	54.8	72	42.9	85	50.6
	*p* value	0.744		0.672		0.499		0.246		0.115	
Workplace	Tertiary govern. hospital	148	59.9	142	57.5	146	59.1	119	48.2	141	57.1
	Private hospital	34	61.8	33	60	31	56.4	23	41.8	30	54.6
	Govern. healthcare center	21	40.4	19	36.5	19	36.5	16	30.8	21	40.4
	*p* value	0.014 *		0.007 *		0.006 *		0.032 *		0.024 *	
Bioethics education	Yes	143	58.6	137	56.2	138	56.6	114	46.7	131	53.7
	No	46	59	45	57.7	46	59	36	46.2	48	61.5
	*p* value	0.976		0.954		0.909		0.908		0.551	
Years of experience	<5	38	61.3	36	58.1	34	54.8	27	43.6	33	53.2
	5–10	52	58.4	49	55.1	50	56.2	41	46.1	48	53.9
	>10	108	56.3	104	54.2	106	55.2	84	43.8	104	54.2
	*p* value	0.702		0.807		0.982		0.917		0.971	

* Independent samples *t*-test/ANOVA is significant at α = 0.05.

**Table 6:** Attitude towards secondary ethical principles (Part B) across demographic data.

Characteristics		Sharing of Benefits		Protecting Future Generations		Protection of the Environment		Respecting Privacy of People		Respecting Confidentiality	
		N	%	N	%	N	%	N	%	N	%
Sex	M	76	58.5	72	55.4	71	54.6	82	63.1	80	61.5
	F	140	54.7	120	46.9	123	48.1	157	61.3	152	59.4
	*p* value	0.456		0.106		0.209		0.704		0.65	
Age (years)	<30	42	59.2	35	49.3	40	56.3	46	64.8	46	64.8
	30–39	80	57.1	72	51.4	69	49.3	91	65	86	61.4
	39–49	58	55.8	53	51	55	52.9	65	62.5	61	58.7
	>49	37	51.4	33	45.8	31	43.1	38	52.8	40	55.6
	*p* value	0.691		0.832		0.367		0.286		0.575	
Married	Yes	160	55	140	48.1	140	48.1	176	60.5	171	58.8
	No	55	58.5	51	54.3	53	56.4	60	63.8	58	61.7
	*p* value	0.763		0.417		0.325		0.752		0.74	
Nationality	Saudi	130	61	113	53.1	112	52.6	144	67.6	136	63.9
	Others	85	50.6	79	47	82	48.8	94	56	96	57.1
	*p* value	0.041 *		0.243		0.465		0.02 *		0.183	
Workplace	Tertiary govern. hospital	143	57.9	128	51.8	131	53	160	64.8	154	62.4
	Private hospital	31	56.4	26	47.3	26	47.3	36	65.5	35	63.6
	Govern. healthcare center	23	44.2	20	38.5	19	36.5	24	46.2	25	48.1
	*p* value	0.045 *		0.126		0.012 *		0.007 *		0.048 *	
Bioethics education	Yes	137	56.2	121	49.6	121	49.6	152	62.3	146	59.8
	No	47	60.3	42	53.9	42	53.9	51	65.4	51	65.4
	*p* value	0.806		0.717		0.47		0.929		0.77	
Years of experience	< 5	35	56.5	28	45.2	31	50	39	62.9	40	64.5
	5–10	48	53.9	45	50.6	45	50.6	56	62.9	54	60.7
	>10	107	55.7	95	49.5	93	48.4	117	60.9	113	58.9
	*p* value	0.949		0.768		0.892		0.879		0.66	

* Independent samples *t*-test/ANOVA is significant at α = 0.05.

**Table 7:** Mean values of the attitude and satisfaction towards ethical issues and attitude towards ethical principles.

Characteristics		Attitude towards Ethical Issues in OB/GYN			Satisfaction towards Ethical Issues in OB/GYN			Attitude towards Ethical Principles in OB/GYN		
		Mean	SD	*p*	Mean	SD	*p*	Mean	SD	*p*
Gender	Male	56	36.7	0.367	43.7	32.7	0.001 *	58.2	27.6	0.612
	Female	59.4	31.8		29.3	31.6		56.7	27	
Age	<30	51.1	36.4	0.191	18.6	24.7	0.001 *	59.2	26.2	0.171
	30–39	59.6	31.7		29.7	29.7		59.2	27.1	
	40–49	61.9	32.3		41.2	35		58	27.9	
	≥50	57.6	34.6		48.6	33.7		51	26.9	
Marital status	Married	57.7	33.2	0.053	39.5	32.9	0.001 *	56.3	27	0.476
	Not married	59.4	33.7		17.2	24.8		59.2	28.1	
Current workplace	Tertiary govern. hospital	64.8	28.1	0.002 *	33.5	33.3	0.001 *	58.8	26.6	0.007 *
	Private hospital	71.3	27.5		49.5	35		58.5	27.8	
	Govern. healthcare center	52.3	33.2		31.2	30		48.5	26.7	
Nationality	Saudi	58.6	33.1	0.984	30.4	31.4	0.009 *	59.2	27.1	0.203
	Non-Saudi	58.6	33.7		39.2	33.6		55.6	27.3	
Bioethics education	Yes	71.3	21.8	0.001 *	37.6	35.3	0.861	58.4	27.6	0.696
	No	63.7	23.6		34.1	34.1		57.4	26	
Experience	<5	52.9	29.8	0.001 *	22.9	30.5	0.001 *	58	27	0.798
	5–10	64.5	27.8		27	29.9		58.7	27.2	
	>10	68.7	28		44.1	34.9		56.5	27.3	

* Independent samples *t*-test/ANOVA is significant at α = 0.05.

## Data Availability

Parts of the datasets generated during the study are available from the corresponding author upon reasonable request.
